# Aggregation and Gelation of Aromatic Polyamides with Parallel and Anti-parallel Alignment of Molecular Dipole Along the Backbone

**DOI:** 10.1038/srep39124

**Published:** 2016-12-13

**Authors:** Dan Zhu, Jing Shang, Xiaodong Ye, Jian Shen

**Affiliations:** 1 Jiangsu Key Laboratory and Bio-functional Materials, School of Chemistry and Materials Sciences, Nanjing Normal University, Nanjing, Jiangsu 210023, China; 2 Hefei National Laboratory for Physical Sciences at the Microscale, Department of Chemical Physics, University of Science and Technology of China, Hefei, Anhui 230026, China

## Abstract

The understanding of macromolecular structures and interactions is important but difficult, due to the facts that a macromolecules are of versatile conformations and aggregate states, which vary with environmental conditions and histories. In this work two polyamides with parallel or anti-parallel dipoles along the linear backbone, named as ABAB (parallel) and AABB (anti-parallel) have been studied. By using a combination of methods, the phase behaviors of the polymers during the aggregate and gelation, i.e., the forming or dissociation processes of nuclei and fibril, cluster of fibrils, and cluster-cluster aggregation have been revealed. Such abundant phase behaviors are dominated by the inter-chain interactions, including dispersion, polarity and hydrogen bonding, and correlatd with the solubility parameters of solvents, the temperature, and the polymer concentration. The results of X-ray diffraction and fast-mode dielectric relaxation indicate that AABB possesses more rigid conformation than ABAB, and because of that AABB aggregates are of long fibers while ABAB is of hairy fibril clusters, the gelation concentration in toluene is 1 w/v% for AABB, lower than the 3 w/v% for ABAB.

Aggregation and gelation of macromolecules are universal and crucial critical phenomena. Aggregation is a kind of spontaneous behavior that produces orders of a system, including crystallization, adsorption, micro- or meso- phase transition and phase separation. It is based on the collective and cooperative non-covalent interactions of molecules, helping us in understanding the highly ordered assembly and immigration phenomena occurred in polymeric materials, and illuminating to fabricate new materials or devices with unique functions. For examples, the aggregation of conjugated polymers or oligomers greatly influences the optical and/or electrical properties of the opto-electronic devices^1^,^2^,^3^, and the aggregate of amyloidogenic protein/peptide attributes to the cause of many transmissible neurogenerative diseases such as Alzheimer’s, Parkinson’s and Huntington’s^4^,^5^,^6^, although the mechanism and kinetics of these chain misfolding, nucleation and aggregation remains largely unknown. Gel is a solid polymer network spanning in the volume of a liquid medium, and the network of a gel is formed through the aggregation of polymer chains or clusters^7^,^8^.

If aggregation or gelation is studied, the intermolecular interaction is inevitably considered. There are usually two ways to understand such phase behaviors. One is from the view of statistical thermodynamics, the driven force of self-assembly comes from the entropy or enthalpy gain or loss. The other is from microscopic level that assembly behavior is usually associated with the intermolecular interactions. The non-covalent inter- or intra- molecular interactions are usually divided into two groups, one is the Columbic force between ions, the other is van der Waals force between dipoles or induced dipoles, both are modified with temperature and medium. Hydrogen bond is usually considered as a unique van der Waals force, it is an electrostatic dipolar-dipolar attraction, but not a covalent bond as termed^9^,^10^. It possesses some features of the covalent bonding, stronger than van der Waals force, and with directional and limited interaction partner. For a long period, it is difficult to distinguish H bonding from the van der Waals forces, including the intrinsic dipolar interaction or the thermally activated induced dipolar interactions. The quantitative description of the dynamics of molecule shall be considered too, because the reversible non-covalent interaction, with the observable characteristic time of 10^−3^~10^2^ s and the Arrhenius life time of 40–80 kJ/mol^11^, is much larger than the thermal energy.

To develop the understanding of the microscopic mechanism of aggregation and gelation of macromolecules and the relevant intermolecular interactions, we characterized two aromatic polyamides with same polar monomer unit, but the dipoles aligned along the backbone are either parallel or anti-parallel, the former is termed as ABAB while the latter is termed as AABB, as shown in Fig. 1. We have visually observed clouding and gelation for both ABAB and AABB in toluene with decreasing the temperature from 80 °C to 20 °C. ABAB demonstrates the clouding point at 60 °C while AABB at 40 °C. The gelation concentrations at r.t. for ABAB and AABB are ca. 3 w/v% and 1 w/v% respectively. We have used differential scanning calorimetry (DSC), transmission electron telescope (TEM) and dielectric relaxation spectroscopy to illuminate the microscopic morphology and dynamics in the processes of aggregation and gelation, focusing on the effect of structural difference in dipolar segmental alignment and orientation, as well as the effect of solvent and temperature, on the aggregation and gelation. We intend to find out the intermolecular interactions dominating in the transition processes. Namely, we would define and describe the size and morphology of the aggregate, the gelation temperature (at constant concentration) or gelation concentration (at constant temperature), the kinetics of aggregation and gelation, and the activation energy involved.

## Experimental

### Materials and Preparation

The synthesis of the ABAB and AABB is normally the polycondensation of polyamides^12^,^13^. The monomers of ABAB were synthesized by following the literatures^14^,^15^,^16^,^17^. The diamine monomers of AABB (monomer-1) and the dicarboxylic acids monomers of AABB (monomer-2) were prepared through the principles in published works^18^,^19^. The FTIR spectra of the monomers and polymers of ABAB and AABB have been shown in the supplementary information.

### Characterizations

The infrared spectra were taken by a Tensor 27 Fourier Transform Infrared Spectrometer (FTIR, Bruker, Germany). Samples were ground, mixed with potassium bromide with weight concentration about 2–3%, and compressed to pellets for testing. Proton nuclear magnetic resonance (^1^H NMR) with an AVANCE 400 spectrometer (Bruker, Germany) was used to verify the prepared polymers, and chloroform-d was used as the solvent. The morphology of ABAB and AABB was observed by using transmission electron microscopes (TEM, JEM-2100F, JOEL, Japan). The samples were prepared with toluene solution of ABAB and AABB (0.01 wt. %) dripped onto the copper grid (400 mesh) coated with carbon film, after thermally treated at temperatures of 60 °C, 40 °C and 20 °C respectively.

The sedimentation coefficient and molar mass were measured via the sedimentation velocity experiments conducted on a Proteomelab XL-A analytical ultracentrifuge (Beckman Coulter Instruments) at 20 °C with an An-60 Ti rotor at the rotational speed of 56 000 rpm. The moving boundary was monitored by repetitive radial scanning at a constant step size of 0.003 cm at 300 nm by using a UV absorption optical system. Sedimentation velocity data were analyzed and simulated by using the software SEDFIT (National Institutes of Health, Bethesda, MD). The continuous c(s) distribution model with maximum entropy regulation was used to obtain s, R_h_ and M_w_, and M_n_ was obtained by c(s, ff0) model. The detailed principle of AUC can be found elsewhere^20^,^21^,^22^.

The differential thermal analysis was performed with a high sensitive differential scanning calorimetry, VP-DSC, Microcal Co., USA. The sensitivity of VP-DSC is 0.2 μW and the resolution is 0.02 μcal/s. It can precisely detect the thermal flow of a solid or liquid sample at a constant temperature. Because there is a pressure perturbation accesory attached to VP-DSC, the liquid or solution samples can be measured too. The samples of ABAB and AABB in toluene were annealed at 80 °C, either fast cooled at a rate of 5 °C/min or slow cooled at 1.5 °C/min, the endothermic curves were recorded during the heating process at heating rate of 1.5 °C/min from 5 °C to 90 °C.

The X-ray diffraction (XRD) characterizations were estimated by a 12 kW rotating-anode X-ray generator (D/max 2500/PC, Rigaku) operated at 150 mA and 40 kV. The samples were dried gel films from toluene solution of ABAB and AABB, casted onto the glass plate layer by layer. Particle sizer (Zetasizer Nano ZS90) from Malvern based on the dynamic LLS principle is used to estimate the size distribution of the polymer aggregate at the scattering angle of 90 °.

Sol or gel samples with different concentration in toluene were tested to characterize their dielectric and electric behaviors at temperature range from 5 °C to 80 °C and frequency range from 10^−2^ to 20 MHz. A broadband dielectric spectrometer (Novocontrol BDS40) was used with a ZGS extension test interface. The applied filed is AC voltage 1.5 Vrms, namely electric filed 750 Vrms/m when the sample cell with thickness of 2 mm was used. The temperature is adjusted with a temperature-control water bath and circulating system with range of 0–60 °C and accuracy of ±0.05 °C.

## Results and Discussion

**Figure 2:**
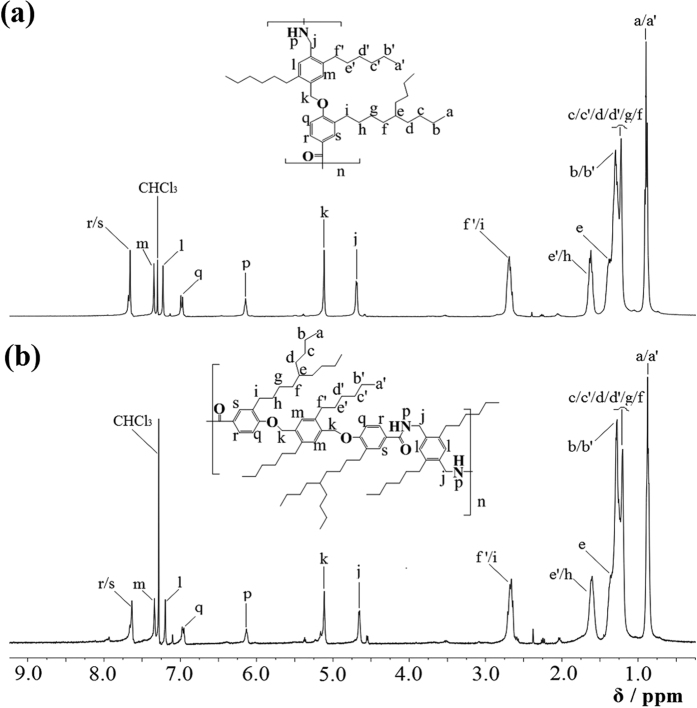
The ^1^H NMR spectra of (**a**) ABAB and (**b**) AABB in chloroform-d.

The experimental ^1^H NMR spectra of the two polymers o ABAB and AABB, which have been taken in the solvent of chloroform-d (CDCl_3_) in the concentration of 1%, are shown in Fig. 2. The chemical structures of ABAB and AABB have been drawn and the chemical shifts of the protons in the polymers have been predicted with ChemDraw and MestReNova, the predicted data has been shown in the supplementary information. The experimental spectra is in good agreement with the predicted in both the chemical shifts and the integral numbers of protons.

**Figure 3:**
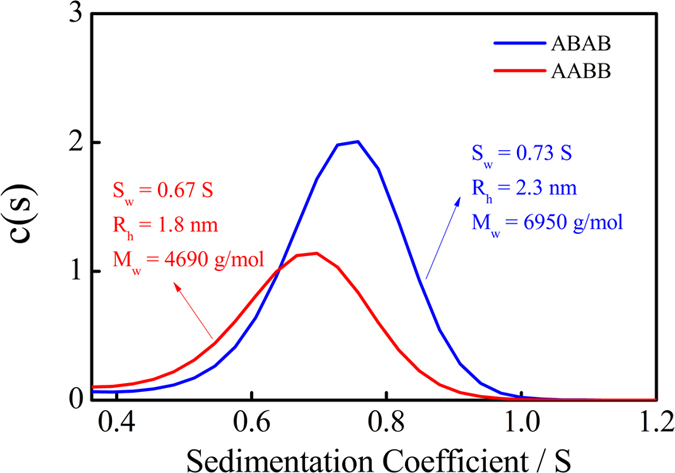
The sedimentation velocity distribution calculated from a SV experiment and Lamm equation analyzed by the c(s) model, for ABAB and AABB in THF.

The analytical ultracentrifuge (AUC) has been used to study the sedimentation velocity (SV) of the two polymers in THF with the concentration of 1.0 mg/ml, to determine the molecular weight of the prepared ABAB and AABB. The principle and original data have been provided in the supplementary information. From the SV test, the sedimentary coefficient (s), the hydrodynamic radius (r) and the weight average molecular weight (*M*w) have been shown in Fig. 3. From the simulation of c(s, ff0) model the weight-average molecular weight (Mw), The number-average molecular weight (Mn), degree of polymerization (DP), and the polydispersity index (Mw/Mn) have been obtained and listed in Table 1.

It has been observed that the polymers can dissolve in solvents of THF, DMSO, and chloroform, but partially dissolve and produce cloudy solvents in toluene, nitrobenzene, styrene *et al*. If polymer concentration is high enough, “gelation” appears in those solvents, because polymer chains have self-organized to form a three-dimensional network and solvent molecules are trapped inside^23^,^24^. Table 2 shows the solubility and gelation concentration of the polymers of ABAB and AABB in the organic solvents.

The solubility of a polymer in a solvent will be determined by their difference in solubility parameters, the less the difference, the higher the solubility. According to Hansen’s pioneering work (1967, 1969)^24^, the solubility parameter δ of either a polymer or a solvents composed of three parts, namely, δ^2^ = δ_d_^2^ + δ_p_^2^ + δ_H_^2^, where δ is the Hildebrandt solubility parameter, δ_d_ represents the part of dispersion forces (van der Waals force), δ_p_ is the part due to dipolar forces, and δ_H_ is the contribution of hydrogen bonding and other donor-acceptor interactions. To predict whether a polymer can be dissolved in a solvent, the sum of the square of the difference in each kind of solubility parameters of the polymer and the solvent shall be calculated, the less the sum, the more the polymer will dissolve in the solvent. Comparing the solubility parameters of THF and toluene, the δ_d_ are almost the same, but the δ_p_ and δ_H_ for THF are higher than those of toluene, we assume the polar and H-bonding interactions between the solvent and the polymer is higher, so that the polymers can dissolve better in THF than in toluene.

We have estimated the three solubility parameters, δ_d_, δ_p_ and δ_H_ for the polymers too, by considering the contributions of molar attractive force of each functional group of the polymers^25^. The solubility parameters δ_d_, δ_p_ and δ_H_ have been derived from the dispersion and polar factors of the attraction constants (F_d_, F_p_), and the H bond energy constant (E_H_) respectively, as shown in Equations 1–3. In the equations *v* is the molar volume. The estimation details can be found in the supplementary information. The dispersion and H bonding parameters, 8.9 and 2.1 Cal^0.5^cm^−1.5^, are the same for ABAB and AABB, but the polarity parameters are 1.0 and 0.7 Cal^0.5^cm^−1.5^ for ABAB and AABB respectively.


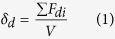



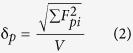



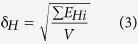


**Figure 4:**
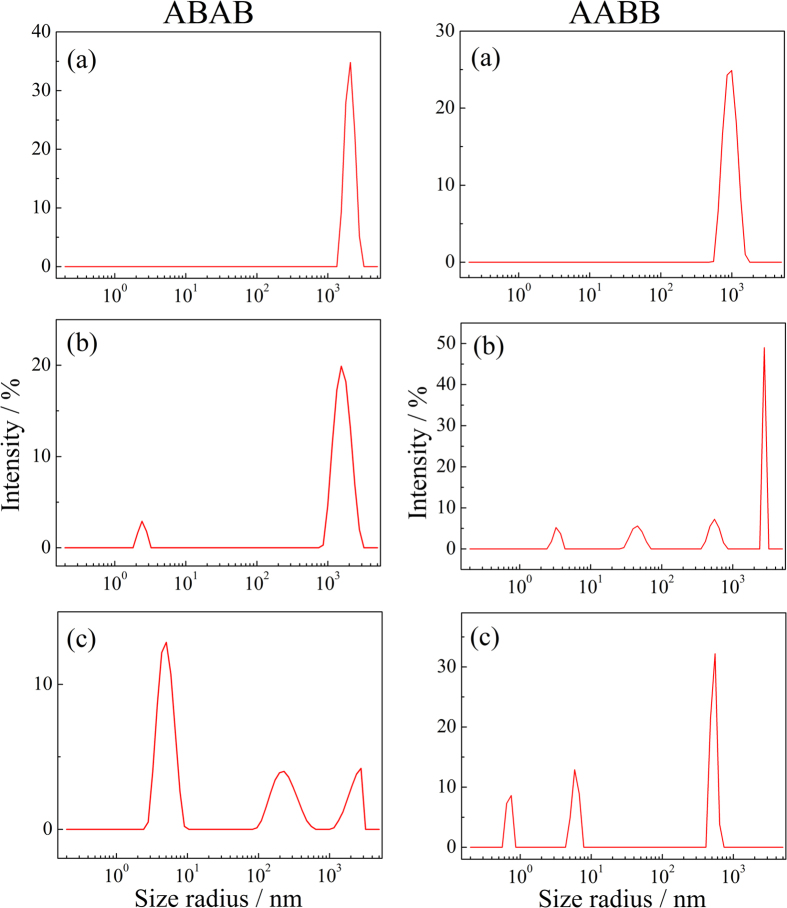
The cluster sizes of ABAB and AABB in toluene, with the concentration of 0.25% w/v, decreases with temperature increasing, (**a**) 25 °C, (**b**) 35 °C, (**c**) 45 °C.

In a good solvent like THF, the solvent with dispersive, polar, and H-bonding provide a strong solubility for the polymers. While in the non-good solvent as toluene, it offers low δ_H,_ the interaction between the polymer and the solvent cannot compete the interactions among the polymers themselves, thus the polymers become to aggregate, and the intermolecular interactions are mostly contributed by H bond interaction. The dispersions of ABAB and AABB in toluene are translucent, indicating the particle size is larger than those in THF. The reason why the particle can sustain a stable size because the alkyl side chains help the polymer in δ_d_ match with organic solvents, the longer and non-symmetric the side chains, the less the δ_d_ difference, the higher the solubility. We have also observed that ABAB is less soluble and more easily aggregate, while AABB is more soluble in toluene and the aggregates are easily disassociate at outer stimuli, such as ultrasonic treatment or shear stress. For the polymer in toluene, increasing the temperature makes the polar and H-bond interaction among the polymer chain decline, and dispersive force enhanced, so that the polymer aggregate dissociates to smaller sizes, which can be observed from the dynamic light scattering measurement. Figure 4 shows the hydrodynamic radius distribution of AABB and ABAB in toluene with the concentration of 0.25% w/v at temperatures of 25 °C, 35 °C and 45 °C, respectively. With increasing the temperatures, the aggregate size decreases for both AABB and ABAB. It is observed that aggregates of AABB dissociate at lower temperature and dissociate to smaller sizes than those of ABAB.

Comparing ABAB and AABB in nitrobenzene or styrene, the factors of δ_H_ are similar between the polymer and solvent, but since the main chains of the polymers are of different symmetry, the δ_p_ differences between the solvent and the polymer are different for ABAB and AABB. In such solvents with similar H bond factor of the polymers, the intermolecular interaction will lie on the polar factors.

**Figure 5:**
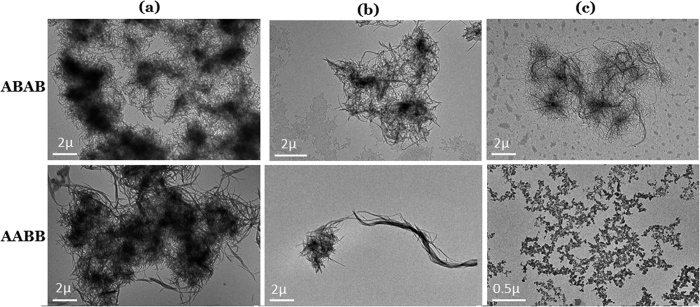
The TEM images of the aggregate morphology of ABAB and AABB, casted from the toluene dispersion at (**a**) room temperature and concentration of 0.05 wt.%, (**b**) room temperature and concentration of 0.005 wt.%, (**c**) 60 °C and concentration of 0.005 wt.%.

Figure 5 shows the images of the cluster or aggregate morphology observed with the transmission electron microscope (TEM), at relatively high and low concentrations and temperatures. The samples have been prepared by casting the toluene dispersion onto the copper grid.

**Figure 6:**
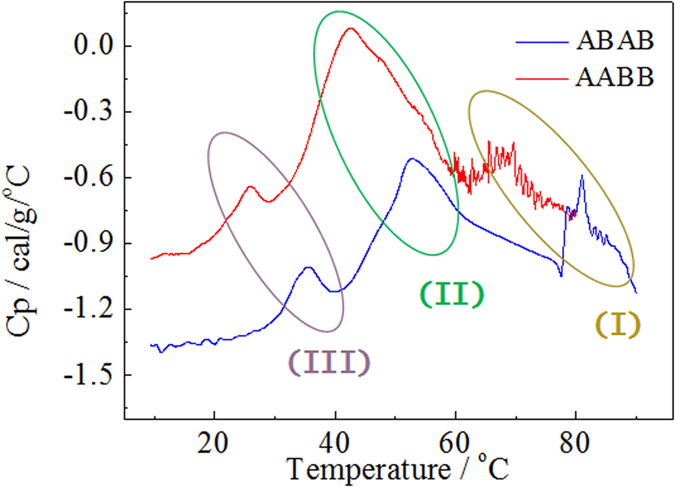
The differential scanning calorimeter curves for ABAB and AABB in toluene with the concentration of 0.5 wt.%.

The ABAB cluster at room temperature looks like a bunch of messy straw, the fibril is thin and short, while in the images of AABB cluster, it is made of longer and thicker fibrils. Gels are formed through the cluster-cluster aggregate, since AABB possesses longer fibrous clusters, it forms a gel at the concentration of 1%, while for ABAB, higher concentration of 3% is necessary at room temperature. Comparing the images taken from the solution at 60 °C, AABB has disassembled into tiny aggregates while ABAB still in the aggregate morphology of fibers or clusters. The tiny aggregates of AABB show the fractal morphology, which has been observed in the aggregation and fibril formation of natural polyamide of proteins and peptides. The fractal pattern is characteristic of self-similarity in structures, dynamics and reactions, it is considered as the initial stage of nucleation via the studies on the static and dynamic light scattering in terms of the diffusion-limited and reaction-limited cluster-cluster aggregation models^26^,^27^,^28^. In describing the grouping of (colloidal) particles aggregation is of wider range than clustering, any elements gathering can be termed as aggregate, while clustering is defined as grouping of the similar elements informally^29^. The fractal structure observed in the initial aggregate stage of AABB indicate AABB is composed of a flexible 1D polymer and resembles the path of self-avoiding random walk^30^,^31^. The formation of the fibril or filament structures from proto-aggregate of molten globule or fractal remains unclear, though it is assumed to be correlated with lateral association or some degree of structural recognition^32^. In the WAXD counts of the dried gel films of ABAB and AABB from toluene, as shown in the supplementary information, the broad equatorial reflections from the lateral packing of chains^33^,^34^ are detected for AABB with a d-spacing of 8.8 Å but not for ABAB, because ABAB backbone is relatively curled, not as rigid as that of AABB. To explore the aggregate or the dissociation mechanism of both polymers and its relation with the gelation, we use the Micro-DSC with high resolution to record the endothermic processes during heating. The results are shown in Fig. 6.

The endothermic peaks appear around 78–85 °C for ABAB and 60–70 °C for AABB, Band I as termed and judged together with the TEM images below or above those temperatures, are related with the transition from micro-sized fibrils cluster to nano-sized globular or fractal aggregates. There are several sub-processes at this transition, assumed as the disassembly of fibrils to globule or fractal nuclei, and nuclei aggregate to macromolecular monomers or oligomers. At 50–60 °C for ABAB and 40–50 °C for AABB, Band II, which is the clouding point visually observed for the two polymers respectively, are attributed to the dissociation of the clusters. The dissociation of ABAB cluster occurs at higher temperature, it is assumed that the linking strength of ABAB fibril is high because of the interaction among polar chains. There is another endothermic process around 25–35 °C for AABB and ABAB, Band III. It is due to the cluster-cluster aggregation, related with the gelation process, although the samples used for DSC test are of the concentration of 0.5%, lower than the gelation concentrations of both.

**Figure 7:**
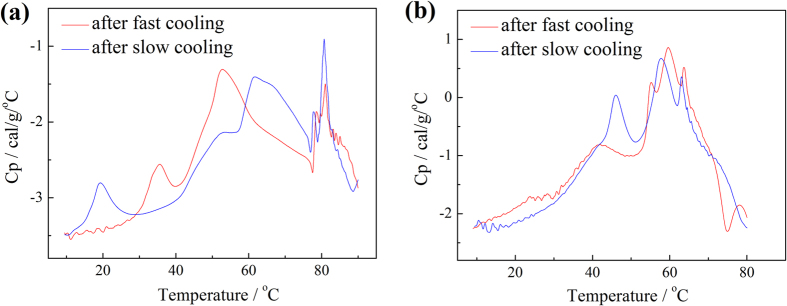
The differential scanning calorimeter curves for (**a**) ABAB and (**b**) AABB in toluene with the concentration about 0.5 wt.%.

The hierarchical structures of a macromolecule are complicated, even if we have performed a perfect synthesis, well determined unit structure and narrow distributed molecular weight, the conformation of the single chain and the condensing of the chain aggregate are of uncertainty on time and heat or stress history. The aggregation and gelation are both thermodynamic and kinetic controlled, such physical gelation is closely related with the packing states of clusters, annealing is carefully performed and thermal history (fast cooling or slow cooling) and heating rate are considered before DSC recording of Fig. 7.

It can be clearly observed there are several sub-processes in Band I, including nucleation, fibril formation and other re-structuring, showing little dependent on the thermal history. Band I of ABAB appear at higher temperature than that of AABB, indicating the nuclei or aggregate of ABAB is more stable. It shows more sub-processes of AABB in Band I than that of ABAB, because AABB shows more morphologies, hairy cluster of tiny fibrils or long thick fibers, as being observed in the TEM images of Fig. 5(b). The Band II of AABB in the first run is broad so that the dissociation of fiber bundles may be merged in. Compare the same polymer quenched at different cooling rates, the cluster dissociation shifts to the low temperatures for the fast cooled samples than that of the slowly cooled. The fast cooling makes the macromolecules freeze at some metastable situation, while the slow cooling offer enough time for the polymer chains to full relaxation and orientation, so that cluster under slow cooling is more stable. After heat treatment, Band III disappears for AABB, while for ABAB annealing at fast cooling rate produces stable cluster-cluster aggregates, suggesting the mechanisms of the aggregations of chains, the clustering of the fibrils and the aggregation of the clusters are different. The fractal morphology indicates that the nucleation of AABB is driven by the non-specific inter-chain interactions, such as hydrogen bonding. The low-temperature process of cluster aggregations is controlled by the van der Waals interactions, which is thermally activated.

**Figure 8:**
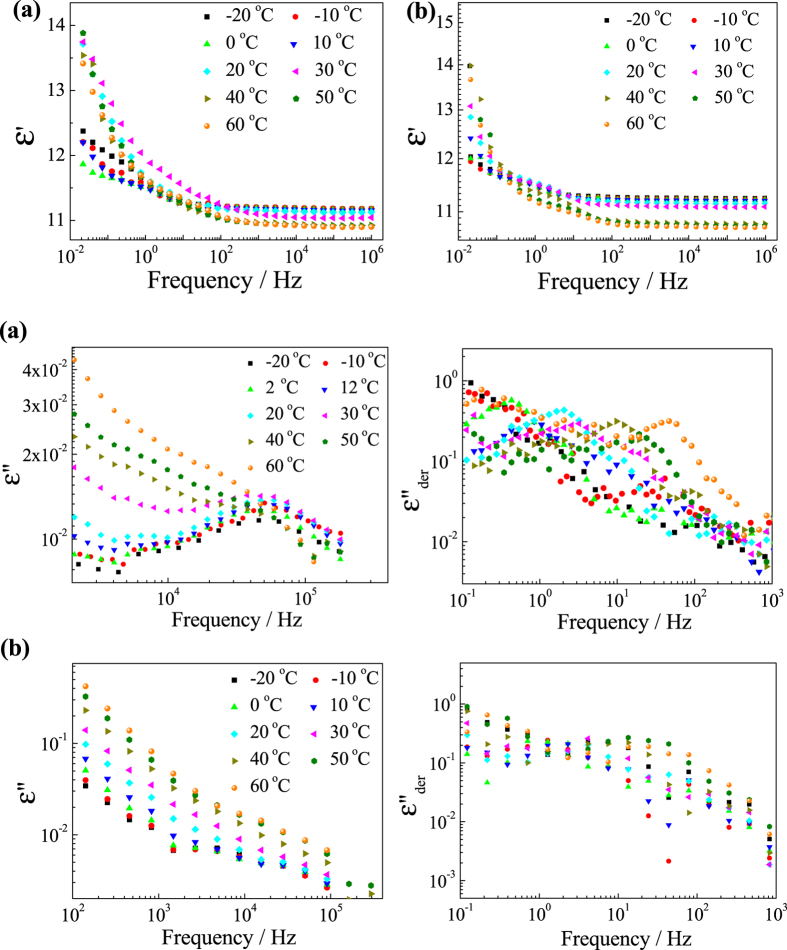
The real parts of the complex permittivity (ε′) and the imaginary parts (ε″) of ABAB (3%) and AABB(1%) in toluene.

Figure 8 shows the real parts of the complex permittivity (ε′) at the range from 0.01 Hz to 1 MHz for ABAB (3%) and AABB (1%) in toluene, and the imaginary parts (ε″) at the high frequency range from1 kHz and 100 kHz (left) and at the low frequency range from 0.1 Hz to 1 kHz (right) for ABAB (3%) and AABB (1%) in toluene. There are two relaxation modes can be observed from the spectroscopy. One is the β-relaxation shown around 6.3 × 10^4^ Hz for ABAB and 1.8 × 10^4^ Hz for AABB respectively, from the location of the maximum permittivity loss. The relaxation time and enhancement of the β-relaxation are almost fixed at the temperature variation. The other is the α-relaxation at 10^0^–10^1^ Hz and is temperature dependent.

The relaxation time of the α-relaxation has been determined from the derivate permittivity loss transformed from the real part by using Kramers-Kronig relation^35^, an effective way to eliminate the Ohmic conduction from the loss at the low frequency range. The fast-mode β-relaxation is attributed to the internal segmental movement of the polymer chains^36^, which is relatively shorter (short relaxation time and maximum loss at high frequency) for ABAB and longer (long relaxation time and maximum loss at low frequency) for AABB, indicating ABAB is less rigidly held, while AABB is in a more expanded conformation. The relaxation increment, i.e. the difference of the real part of the complex permittivities below or beyond the relaxation, is proportional to the amount of the dipolar involved in the relaxation. The β-relaxation of ABAB shows higher increment than that of AABB, since its segmental dipolar moment is obviously larger because of the parallel alignment of the monomers.

**Figure 9:**
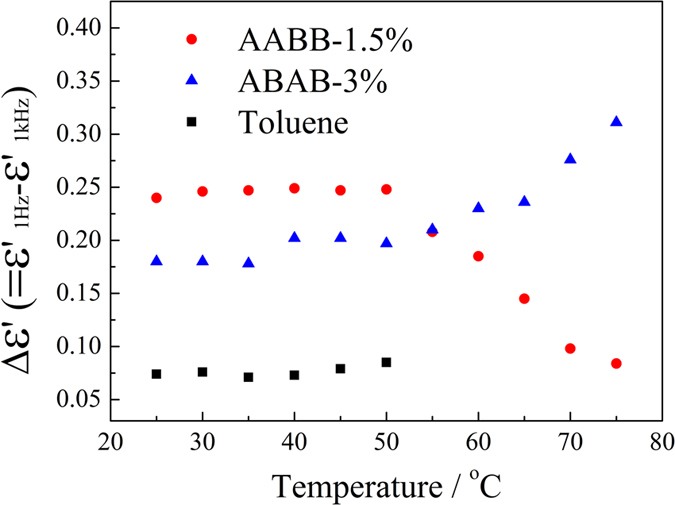
The temperature dependence of relaxation increments of the α-relaxation of ABAB (1.8%) and (b) AABB(1.5%) in toluene, and that of toluene.

To identify the attribution of the α-relaxation, trials and efforts have been made. It has been observed that the relaxation time increases with the temperature increasing, which is common in most polarizations and helps little in determining the attribution. The relaxation increments of α-relaxation, i.e. the difference of the real part of the complex permittivity between 1 kHz and 1 Hz, have been plotted against temperature, as shown in Fig. 9.

**Figure 10:**
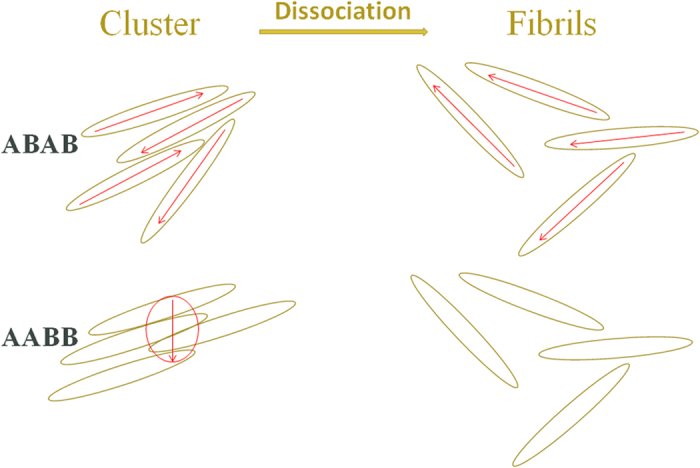
The schematic diagram of the aggregate dissociation of ABAB and AABB, indicating relaxation increments of the α-relaxation of ABAB increases while that of AABB decreases with temperature higher than the clouding point.

Considering the increment of ABAB and AABB change with temperature abruptly above the clouding point, which correlates to the fibrils aggregation and dissociation, the slow-mode α-relaxation is defined as the interfacial polarization^37^,^38^. The interfacial polarization is also termed as the Maxwell-Wagner-Sillars polarization, which is generated because dielectric effects generated by the applied field on the polymer phase and solvent phase do not match or cancel with each other. Let us assume the polymer fibrils in the solvent media behave like huge dipoles, the increment equals to the cumulative sums of dipolar moment vectors in a unit volume. It shows in Fig. 9 that the increment of AABB decreases and that of ABAB increases with temperature when the temperature is higher than the clouding points. As shown in the schematic diagram of Fig. 10, the cluster dissociation occurs at temperature higher than the clouding point. When ABAB clusters segregate, there are more dipolar fibrils are liberated and contributes to the bulk polarity. In separated AABB fibrils, the dipoles are none because of the anti-parallel alignment. While in the clusters of AABB, mostly in fibril bundles because of the rigidity of the AABB chains, there are the dipolar domains which contributes to the polarity, so that the relaxation increment decreases when the bundles get dissociated. In the frequency dependent complex permittivity (real and imaginary parts) of the power form polymers pressed into a tablet, as shown in the supplementary information, the permittivity of AABB is about 24, 3–4 time higher than that of ABAB, which indicates there are highly polarizable domains in the aggregates of AABB.

## Conclusions

In conclusion, we have successfully characterized two aromatic polyamides with dipoles parallel or anti-parallel along the linear backbone. The structures have been defined via FTIR, ^1^H NMR and AUC. It has been observed aggregation and gelation for both polymers in toluene, styrene and nitrobenzene at temperature decreasing from 80 °C to 20 °C. DLS and TEM detects the size and morphology change of ABAB and AABB in toluene with temperatures, it discloses the AABB aggregates are long and thick fibers while ABAB is of hairy fibril clusters. The DSC and DRS have recorded the dissociation processes of the clusters’ aggregations, clusters into fibrils, and the fibrils into nuclei of macromolecules with the temperature increasing. It has revealed abundant phase behaviors of the two polymers in the non-good solvent, and the intermolecular interaction has been found to be correlated with the solubility parameters of solvents, the temperature, and the polymer concentration.

## Additional Information

**How to cite this article**: Zhu, D. *et al*. Aggregation and Gelation of Aromatic Polyamides with Parallel and Anti-parallel Alignment of Molecular Dipole Along the Backbone. *Sci. Rep.* **6**, 39124; doi: 10.1038/srep39124 (2016).

**Publisher's note:** Springer Nature remains neutral with regard to jurisdictional claims in published maps and institutional affiliations.

## Supplementary Material

Supplementary Information

## Figures and Tables

**Figure 1:**
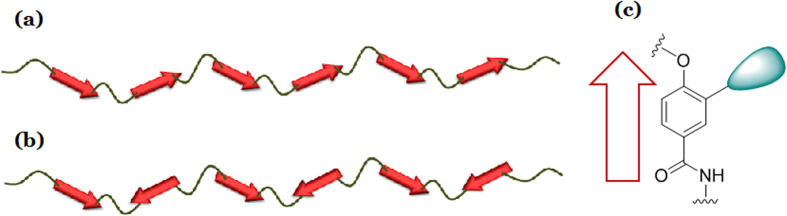
Schematic structures of (**a**) ABAB with parallel dipoles along the backbone, (**b**) AABB with anti-parallel dipoles along the backbone. It shows in (**c**) the chemical structure of the monomer furnishing dipolar moment.

**Table 1:** The average molecular weights and polydispersity index of ABAB and AABB determined by AUC.

Sample	Mw (g/mol)	DP	Mn (g/mol)	PDI
ABAB	6320	11	4110	1.5
AABB	4890	4	3740	1.3

**Table 2:** The solubility and gelation concentration of ABAB and AABB in organic solvents, the solubility parameters of the solvents are listed.

	Solubility of polymer and critical gel concentration at r.t.		Solubility parameters of solvents (Cal^1/2^cm^−3/2^)		
	ABAB	AABB	δ_d_	δ_p_	δ_H_
THF	soluble	soluble	8.21	2.79	3.91
DMSO	soluble	soluble	9.00	8.02	4.99
CHCl_3_	soluble	soluble	8.80	1.52	2.59
Toluene	gel-3%	gel-1%	8.80	0.68	0.98
Nitrobenzene	gel-2%	gel-5%	9.78	4.2	2.00
Styrene	gel-3%	gel-2.5%	9.07	0.49	2.00

## References

[b1] Blatchford J. W. . Spatially and temporally resolved emission from aggregates in conjugated polymers. Physical Review B Condensed Matter. 54, 3683–3686 (1996). 10.1103/physrevb.54.r3683 9986346

[b2] Wang D., Yuan Y., Mardiyati Y., Bubeck C. & Koynov K. From single chains to aggregates, how conjugated polymers behave in dilute solutions. Macromolecules. 46, 6217–6224 (2013).

[b3] Yao Y., Dong H. & Hu W. Ordering of conjugated polymer molecules: recent advances and perspectives. Polymer Chemistry. 4, 5197–5205 (2013).

[b4] Pappu R. V., Wang X., Vitalis A. & Crick S. L. A polymer physics perspective on driving forces and mechanisms for protein aggregation. Archives of Biochemistry & Biophysics. 469, 132–141 (2008). 17931593 10.1016/j.abb.2007.08.033 PMC2211569

[b5] Paulson H. L., Bonini N. M. & Roth K. A. Polyglutamine disease and neuronal cell death. Proceedings of the National Academy of Sciences. 97, 12957–12958 (2000). 10.1073/pnas.210395797 PMC34075 11058149

[b6] Giurleo J. T., He X. & Talaga D. S. β-lactoglobulin assembles into amyloid through sequential aggregated intermediates. Journal of Molecular Biology. 381, 1332–1348 (2008). 18590743 10.1016/j.jmb.2008.06.043

[b7] Coniglio A., Dearcangelis L., Delgado E., Fierro A. & Sator N. Percolation, gelation and dynamical behaviour in colloids. Journal of Physics Condensed Matter. 16, 4831–4839 (2004).

[b8] Herrmann J. Kinetics of Aggregation and Gelation (ed. Family F., Landau D. P.) (Elsevier, 1984).

[b9] Jeffrey G. A. An Introduction to Hydrogen Bonding (ed. Donald G. T.) (Oxford University Press, 1997).

[b10] Steiner T. The hydrogen bond in the solid state. Angewandte Chemie International Edition. 41, 48–76 (2002). 10.1002/1521-3773(20020104)41:1<48::aid-anie48>3.0.co;2-u 12491444

[b11] Jones R. A. L. & Weitz D. A. Forces, energies, and timescales in condensed matter In Soft Condensed Matter. 5–16 (Oxford University Press, 2002).

[b12] Ueda M., Morishima M., Kakuta M. & Sugiyama J. Synthesis of ordered polyamides by direct polycondensation. 3. Macromolecules. 25, 6580–6585 (1992).

[b13] More A. S., Pasale S. K. & Wadgaonkar P. P. Synthesis and characterization of polyamides containing pendant pentadecyl chains. European Polymer Journal. 46, 557–567 (2010).

[b14] Lee J. C., Yuk J. Y. & Cho S. H. Cheminform abstract: facile synthesis of alkyl phenyl ethers using cesium carbonate. Synthetic Communications. 25, 1367–1370 (1995).

[b15] Hofsløkken N. U. . Convenient method for the ortho-formylation of phenols. Acta Chemica Scandinavica. 53, 258–262 (1999).

[b16] Wittig G. & Schöllkopf U. Über Triphenyl-phosphin-methylene als olefinbildende reagenzien (I. Mitteil.). Chemische Berichte. 87, 1318–1330 (1954).

[b17] Wittig G. & Haag W. Über Triphenyl-phosphin-methylene als olefinbildende reagenzien (II. Mitteil.). Chemische Berichte. 88, 1654–1666 (1955).

[b18] Scriven E. F. V. & Turnbull K. Azides: their preparation and synthetic uses. Chemical Reviews. 88, 297–368 (1988).

[b19] Hunt I. & Spinney R. Organic Chemistry On-Line Learning Center, http://www.chem.ucalgary.ca/courses/350/Carey5th/Carey.html (2006).

[b20] Schuck P. Size-distribution analysis of macromolecules by sedimentation velocity ultracentrifugation and lamm equation modeling. Biophysical Journal. 78, 1606–1619 (2000). 10692345 10.1016/S0006-3495(00)76713-0 PMC1300758

[b21] Luo Z., Wang X. & Zhang G. Ion-specific effect on dynamics of polyelectrolyte chains. Physical Chemistry Chemical Physics. 14, 6812–6816 (2012). 22495384 10.1039/c2cp40077d

[b22] Luo Z. & Zhang G. Sedimentation of polyelectrolyte chains in solutions: from dilute to semidilute. Polymer. 52, 5846–5850 (2011).

[b23] Almdal K., Dyre J., Hvidt S. & Kramer O. Towards a phenomenological definition of the term ‘gel’. Polymer Gels & Networks. 1, 5–17 (1993).

[b24] Coniglio A., De Arcangelis L., Del Gado E., Fierro A. & Sator N. Percolation, gelation and dynamical behaviour in colloids. Journal of Physics Condensed Matter. 16, 4831–4839 (2004).

[b25] Van Krevelen D. W. & Te Nijenhuis K. Properties of Polymers: their correlation with chemical structure: their numerical estimation and prediction from additive group contributions. 189–227 (Elsevier, 2009).

[b26] Chen B., Nellas R. B. & Keasler S. J. Fractal aggregates in protein crystal nucleation. Journal of Physical Chemistry B. 112, 4725–4730 (2008). 10.1021/jp8002728 18358033

[b27] Fadda G. C. & Lairez D. Rigid structure of fractal aggregates of lysozyme. Europhysics Letters. 52, 712–718 (2000).

[b28] Umbach P., Georgalis Y. & Saenger W. Time-resolved small-angle static light scattering on lysozyme during nucleation and growth. Journal of the American Chemical Society. 120, 2382–2390 (1998).

[b29] Gionis A., Mannisla H. & Tsaparas P. ACM Transactions on Knowledge Discovery from Data. *dblp*. volume 1, Article No. 4 (2007).

[b30] Gerstman B. S. & Chapagain P. P. Self-organization dynamics in protein folding In Molecular Biology of Protein Folding (ed. Conn P. M.) Progress in Molecular Biology and Translational Science, Part B, 84, 1–37 (Academic Press, 2008). 10.1016/S0079-6603(08)00401-7 19121698

[b31] Pappu R. V., Wang X., Vitalis A. & Crick S. L. A polymer physics perspective on driving forces and mechanisms for protein aggregation. Archives of Biochemistry & Biophysics. 469, 132–141 (2008). 17931593 10.1016/j.abb.2007.08.033 PMC2211569

[b32] Nimmanpipug P., Tashiro K., Maeda Y. & Rangsiman O. Factors governing the three-dimensional hydrogen bond network structure of poly (m-phenylene isophthalamide) and a series of its model compounds: (1) Systematic classification of structures analyzed by the X-ray diffraction method. The Journal Physical Chemistry B. 106, 6842–6848 (2002). 10.1021/jp062058r 17048899

[b33] Liou G., Hsiao S., Huang N. & Yang Y. Synthesis, photophysical, and electrochromic characterization of wholly aromatic polyamide blue-light-emitting materials. Macromolecules. 39, 5337–5346 (2006).

[b34] Fu K., Sone M., Tokita M. & Watanabe J. Aromatic polyesters with flexible side chains. 10. studies on biaxiality in nematic liquid crystal of bc-n polyester. Polymer Journal. 38, 442–446 (2006).

[b35] Wübbenhorst M. & Turnhout J. V. Analysis of complex dielectric spectra. i. one-dimensional derivative techniques and three-dimensional modelling. Journal of Non-Crystalline Solids. 305, 40–49 (2002).

[b36] Diaham S. & Locatelli M. L. Dielectric properties of polyamide-imide. Journal of Physics D: Applied Phys. 46, 185302 (2013).

[b37] Steeman P. A. M. & van Turnhout J. Broadband dielectric spectroscopy In Dielectric Properties of Inhomogeneous Media (ed. Kremer F., Schonhals A.) 495–522 (Springer Berlin Heidelberg, 2003).

[b38] Brosseau C. Modelling and simulation of dielectric heterostructures: a physical survey from an historical perspective. Journal of Physics D Applied Physics. 39, 1277–1294 (2006).

