# Supplementary material for: Aggregation and Gelation of Aromatic Polyamides with Parallel and Anti-parallel Alignment of Molecular Dipole Along the Backbone
Source: Sci Rep. 2016 Dec 13;6:39124. doi: 10.1038/srep39124 (PMC5153643; doi:10.1038/srep39124)
Supplement: Supplementary Information [file srep39124-s1.pdf]

Supplementary Information

Aggregation and Gelation of Aromatic Polyamides with Parallel and Anti-parallel Alignment of Molecular Dipole Along the Backbone

Dan Zhu[a,*] , *Jing Shang*[a], *Xiaodong Ye*[b] , and *Jian Shen* [a,*]

[a]*Jiangsu Key Laboratory and Bio-functional Materials, School of Chemistry and Materials Sciences, Nanjing Normal University, Nanjing, Jiangsu 210023, China, zhudan@njnu.edu.cn*
[b]*Hefei National Laboratory for Physical Sciences at the Microscale, Department of Chemical Physics, University of Science and Technology of China, Hefei, Anhui 230026, China*

1. FTIR

The FTIR spectra of monomers and polymers of ABAB and AABB have been shown in Figures 1. The N-H and C=O bands have been observed in the spectra, locating at 3300-3400 cm^{-1} and 1650-1700 cm^{-1} respectively as indicated in the graph. Compared with those of the monomers the bands shift to the low wavenumbers for the polymers, indicating the formation of hydrogen bonding in the polymers.

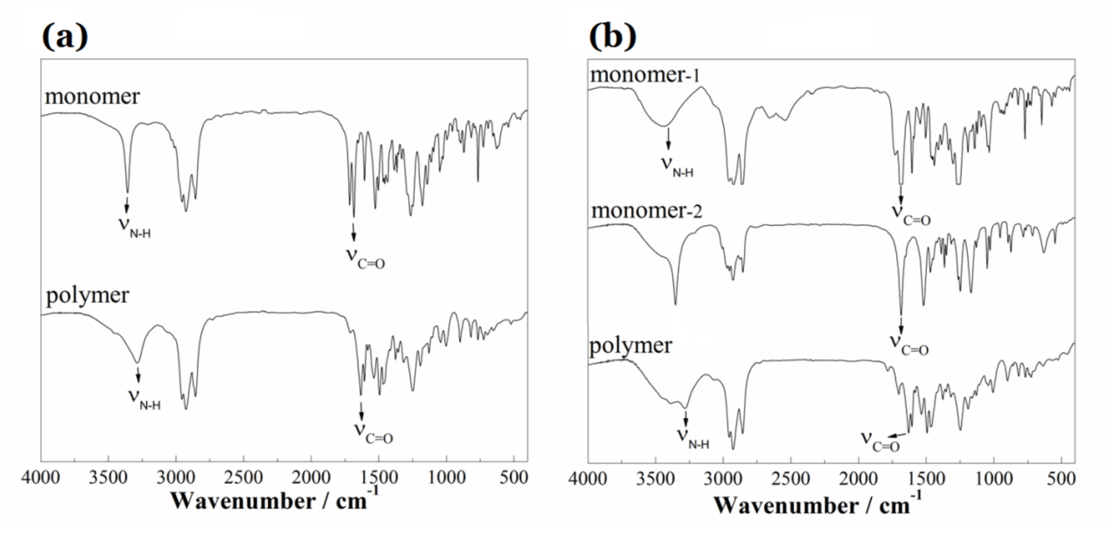

Figure 1. FTIR spectra for monomers and polymers of (a) ABAB and (b) AABB.

2. ^{1}H NMR

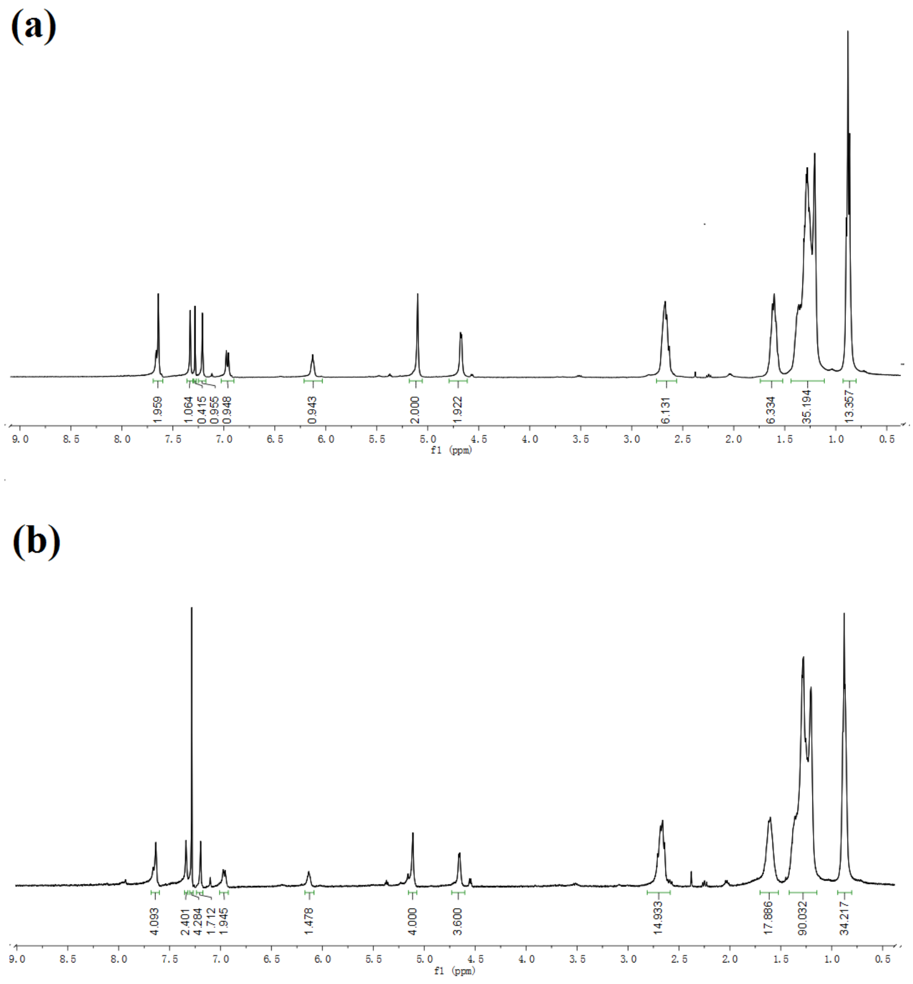

Figure 2. Integral areas of ^{1}H NMR spectra of (a) ABAB and (b) AABB.

The predicted chemical shift of the proton in amine is 8.0 ppm, but in the experimental spectra, it is located at 6.2 because of the polymerization and the hydrogen bonding with carbonyl group. In ABAB the chemical shifts of the protons at the benzene rings, attributed by q, l, m, and r/s protons noted at the structural formula, are mostly located at 7.0, 7.1, 7.3, and 7.7 ppm with the integral proton numbers of 0.9, 0.9, 1.0, and 1.9 respectively, if we assign the number of the k proton at the benzyl group, which is located at 5.16 ppm in the spectroscopy, as 2. The chemical shift at 7.24 ppm with 0.4 proton cannot be attribute to any but has to be considered as the contribution from the trace of undeuterated solvent.

3. AUC

Figure 3 shows the sedimentary velocity results of analytical ultracentrifuge for the prepared ABAB and AABB in THF solutions with the concentration of 1.0 mg/ml.

(a) ABAB

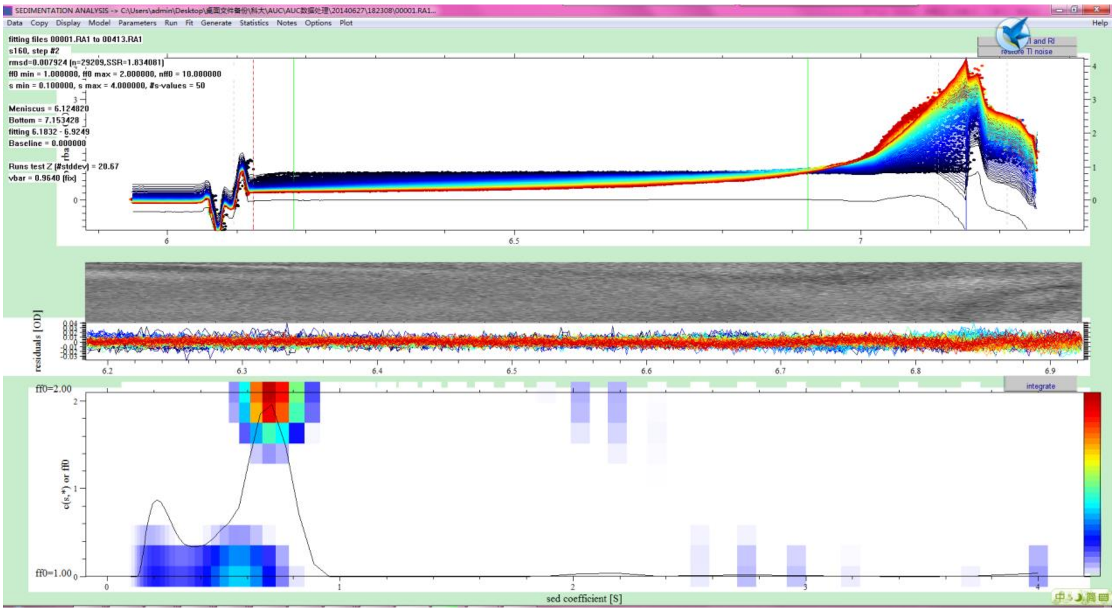

(b) AABB

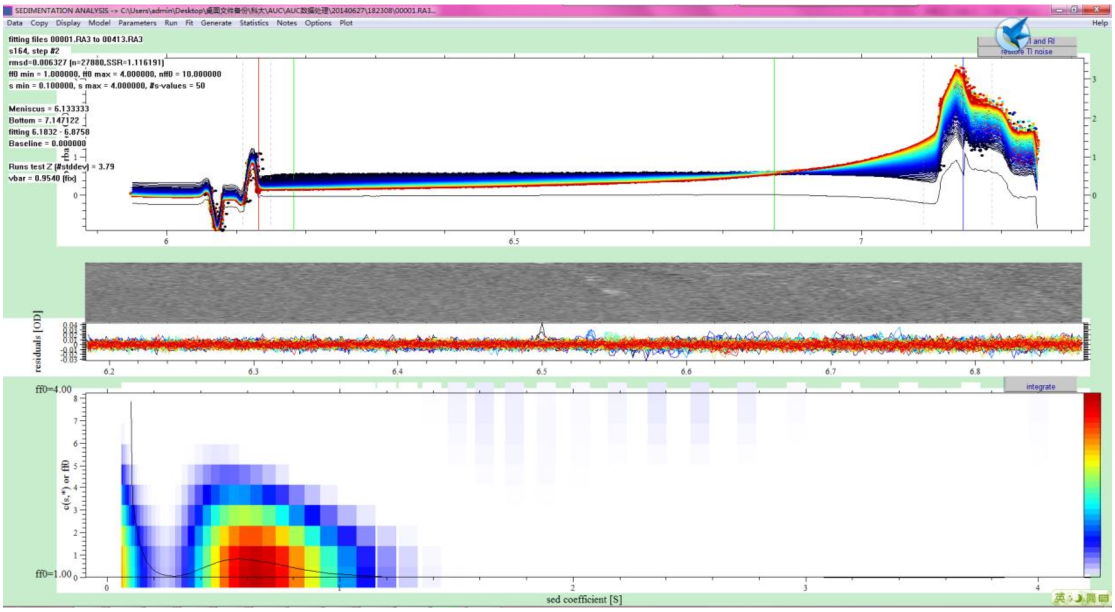

Figure 3. Sedimentary velocity results of analytical ultracentrifuge for the prepared ABAB and AABB in THF solutions with the concentration of 1.0 mg/ml.

The sedimentation process can be described by the Lamm equation

$$\frac{\partial c}{\partial t} = \mathrm{D}\left(\frac{\partial^2 c}{\partial r^2} + \frac{1}{r}\frac{\partial c}{\partial r}\right) - s\omega^2\left(r\frac{\partial c}{\partial r}\right) + 2c$$ **Equation. 1**

where c, t, D, u, r and s are the solute concentration, sedimentation time, diffusion coefficient, angular velocity, radial distance from the rotation axis and sedimentation coefficient in unit of Svedberg (S). The sedimentation coefficient is defined as

$$\mathrm{s} = \frac{u}{\omega^2 r} = \frac{dlnr}{\omega^2 dt}$$ **Equation. 2**

where u is the sedimentation velocity of the solute.

s, D relates to the molar mass (M) by the Svedberg equation

$$\mathrm{M} = \frac{sRT}{D(1-\nu\rho_s)}$$ **Equation. 3**

where M, R, T, ρ_s, υ are the molar mass, gas constant, absolute temperature, solvent density and partial specific volume (v-bar), respectively.

The partial specific volume (v-bar), solvent density and viscosity have been listed in Table 1.

Table 1. The partial specific volume (v-bar), solvent density (ρ_s) and viscosity (η) tested in the AUC experiment.

	ABAB	AABB
v-bar	0.964 ml/g	0.954 ml/g
ρ_s	0.88786 g/ml	0.88786 g/ml
η	0.53 cP	0.53 cP

4. Solubility parameters

We have estimated the three solubility parameters, δ_d, δ_p and δ_H, respectively from the Equations 4-6.

$$\delta_d = \frac{\sum F_{di}}{V},\ \delta_p = \frac{\sqrt{\sum F_{pi}^2}}{V},\ \delta_H = \sqrt{\frac{\sum E_{Hi}}{V}} \qquad \textbf{Equations. 4-6}$$

In which F_d and F_p are the dispersion and polar factors of the attraction constants, and E_H is the H bond energy constant, v is the molar volume.

Table 2. The molar attractive forces and molar volume contributed by each group in the polymers.

	F_{di} $\left(J^{\frac{1}{2}} \cdot cm^{\frac{3}{2}} \cdot mol^{-1}\right)$	F_{pi} $\left(J^{\frac{1}{2}} \cdot cm^{\frac{3}{2}} \cdot mol^{-1}\right)$	E_{Hi} $(J \cdot mol^{-1})$	Molar Volume $(cm^3 \cdot mol^{-1})$
—CH_3	420	0	0	33.5
—CH_2—	270	0	0	16.1
—CH—	80	0	0	-1.0
—C_6H_4—(para)	1270	110	0	52.4
—O—	100	400	3000	3.8
—NH—	160	210	3100	9.5
—CO—	290	770	2000	

Table 3. The estimated solubility parameters of the polymers and those of the solvents referred in literature.

	δ_d $\left(J^{\frac{1}{2}} \cdot cm^{-\frac{3}{2}}\right)$	δ_p $\left(J^{\frac{1}{2}} \cdot cm^{-\frac{3}{2}}\right)$	δ_H $\left(J^{\frac{1}{2}} \cdot cm^{-\frac{3}{2}}\right)$	Molar Volume $(cm^3 \cdot mol^{-1})$
ABAB	18.26	2.12	4.35	428.2
AABB	18.26	1.50	4.35	428.2
Benzene	18.4	0	2.0	89.4
Toluene	18.4	1.4	2.0	106.8
Nitrobenzene	20.1	8.6	4.1	102.7
Styrene	18.6	1.0	4.1	115.6

Note: If the unit of $Cal^{0.5}cm^{-1.5}$ is used, the data shall be divided by 2.05.

5. XRD

Figure 4 shows the WAXD counts recorded for the dried gels of ABAB and AABB, which are obtained from their toluene dispersion at 80 °C and quenched to room temperature. There are diffraction peaks detected for both ABAB and AABB located at 2θ of 4.5° with d-spacing of 19.5 Å. Usually the (001) meridional reflection appears

with a spacing of ~ 17-19 Å corresponds to the average repeat distance of polyamide backbone. The broad equatorial reflections at small angle (inner, 8-9 Å) or wide angle (outer, 4-5 Å) regions result from the lateral packing structures. The outer reflection with the 2θ at 21.4° and the d-spacing of 4.1 Å detected for both polymers corresponds to the lateral packing distance of aromatic molecules, irrelevant with the length of the alkyl side chains. But the inner one, only been detected for AABB at 2θ 10.1° and d-spacing of 8.8 Å, shows the possibility of another order in lateral packing, such as biaxial orientation of molecules. The alkyl side chain is about 10 Å, the lateral packing with d-spacing of 8.8 Å can be achieved by the side-by-side alignment of the side chains of the neibouring polymers in AABB. While in ABAB, such inner reflection is not detected, because the backbone is relatively curled, not as rigid as that of AABB.

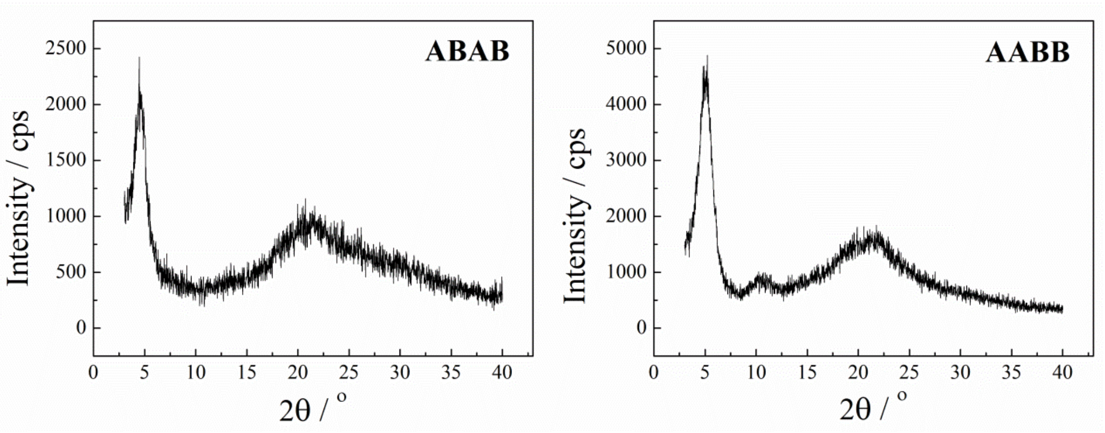

Figure 4. WAXD counts of the dried gel films of ABAB and AABB from toluene.

6. Dielectric spectroscopy of the solid samples

Figure 5 is the frequency dependent complex permittivity (real and imaginary parts) measured for the solid polymers and ABAB and AABB. The polymers in powder form have been pressed into a tablet before testing. It shows in the figure that AABB

possesses 3-4 time higher permittivity than that of ABAB. In dealing with the powder samples, AABB does show higher static electrical property that it adheres to other objects.

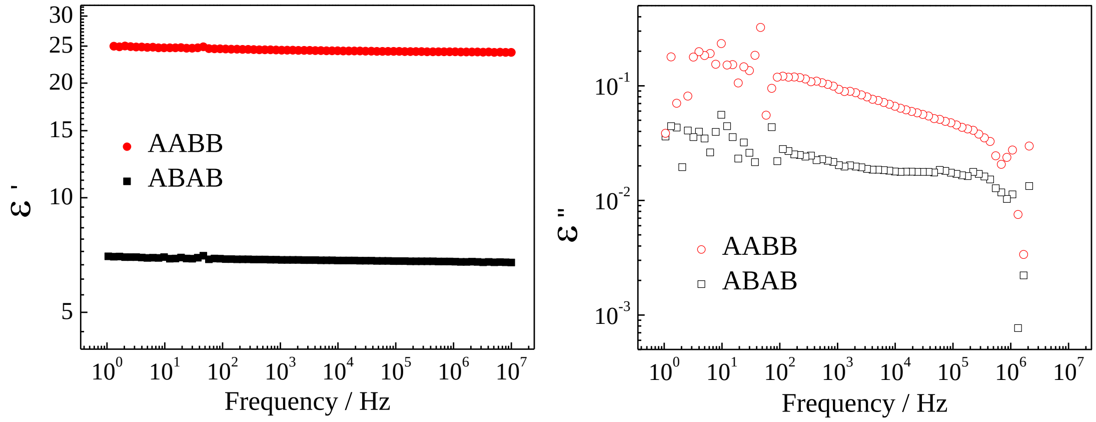

Figure 5. Frequency dependent real and imaginary parts of complex permittivity (ε' and ε") of polymers of ABAB and AABB.
